# Real-time PCR methods for identification and stability monitoring of *Bifidobacterium longum* subsp. *longum* UABl-14 during shelf life

**DOI:** 10.3389/fmicb.2024.1360241

**Published:** 2024-04-19

**Authors:** Hanan R. Shehata, Basma Hassane, Steven G. Newmaster

**Affiliations:** ^1^Purity-IQ Inc., Guelph, ON, Canada; ^2^Department of Integrative Biology, College of Biological Science, University of Guelph, Guelph, ON, Canada; ^3^Department of Microbiology, Faculty of Pharmacy, Mansoura University, Mansoura, Egypt

**Keywords:** real-time PCR, probe-based assay, strain specific PCR assay, probiotics, viability PCR, PMAxx, *Bifidobacterium longum* subsp. *longum* UABl-14, viable but non culturable

## Abstract

*Bifidobacterium longum* subsp. *longum* UABl-14^™^ is an important probiotic strain that was found to support digestive health. Here we present the development and validation of real-time PCR methods for strain-specific identification and enumeration of this important strain. The identification method was evaluated for specificity using 22 target samples and 30 non-target samples. All target samples successfully amplified, while no amplification was observed from any non-target samples including other *B. longum* strains. The identification method was evaluated for sensitivity using three DNA dilution series and the limit of detection was 2 pg. of DNA. Coupled with a viability dye, the method was further validated for quantitative use to enumerate viable cells of UABl-14. The viability dye treatment (PMAxx) was optimized, and a final concentration of 50 μM was found as an effective concentration to inactivate DNA in dead cells from reacting in PCR. The reaction efficiency, linear dynamic range, repeatability, and reproducibility were also evaluated. The reaction efficiency was determined to be 97.2, 95.2, and 95.0% with *R*^2^ values of 99%, in three replicates. The linear dynamic range was 1.3 × 10^2^ to 1.3 × 10^5^ genomes. The relative standard deviation (RSD%) for repeatability ranged from 0.03 to 2.80, and for reproducibility ranged from 0.04 to 2.18. The ability of the validated enumeration method to monitor cell counts during shelf life was evaluated by determining the viable counts and total counts of strain UABl-14 in 18 multi-strain finished products. The viable counts were lower than label claims in seven products tested post-expiration and were higher than label claims in products tested pre-expiration, with a slight decrease in viable counts below label claim in three samples that were tested 2–3 months pre-expiration. Interestingly, the total counts of strain UABl-14 were consistently higher than label claims in all 18 products. Thus, the method enables strain-specific stability monitoring in finished products during shelf life, which can be difficult or impossible to achieve using the standard plate count method. The validated methods allow for simultaneous and cost-effective identification and enumeration of strain UABl-14 and represent an advancement in the quality control and quality assurance of probiotics.

## Introduction

Probiotics are defined as “live microorganisms that, when administered in adequate amounts, confer a health benefit on the host” (Hill et al., 2014). Delivering the correct probiotic strains at the correct dose of viable cells is essential to achieve their health benefits (Tripathi and Giri, 2014; Kolaček et al., 2017; Sánchez et al., 2017). However, several studies reported variable rates of non-compliance in probiotic products, more specifically, failure of probiotic products to meet declared strain contents and/or viable counts (Morovic et al., 2016; Shehata and Newmaster, 2020a,b). Thus, reliable, and accurate methods for probiotic strain identification and viable count determination are essential for probiotic authentication and quality assessment.

PCR based methods are widely used for probiotic identification including species-specific and strain-specific methods (Morovic et al., 2016; Kim et al., 2020; Shehata et al., 2021a,b; Kim et al., 2022). For probiotic enumeration, plate count methods are currently the most commonly used methods for probiotic quantification (Davis, 2014; Weitzel et al., 2021; Boyte et al., 2023), however, other culture-independent methods such as flow cytometry and PCR based methods are also emerging for probiotic enumeration (Boyte et al., 2023).

Plate count methods have several limitations such as the low specificity, i.e., inability to enumerate individual strains in multi-strain blends as these methods enable enumeration at the genus level or species level only if using selective growth media. This is a huge limitation since the health benefits of probiotics are strain specific (Klein et al., 2010; Sánchez et al., 2017; Mcfarland et al., 2018). Furthermore, plate count methods are culture-dependent methods which measure viability as cultivability, and thus these methods do not detect cells that exist in a viable but non culturable (VBNC) state (Wilkinson, 2018; Gorsuch et al., 2019; Wendel, 2022).

Alternative enumeration methods such as flow cytometry and viability PCR based methods are culture-independent methods that measure viability beyond cultivability (ISO, 2015; Hansen et al., 2018, 2020; Foglia et al., 2020; Kim E. et al., 2023; Ma et al., 2023; Shehata et al., 2023). Thus, these methods are able to count VBNC cells, hence, more accurate viable count determination. Additionally, PCR based methods can be designed to achieve strain specific viable count determination (García-Cayuela et al., 2009; Kramer et al., 2009), which is a huge improvement from the traditional plate count methods. PCR methods can be used with viability dyes in what is called viability PCR to quantify viable cells only (Hansen et al., 2020; Shehata et al., 2023). PCR methods are less laborious, high throughput, and offer shorter time to results (~6 h). Given the advantages of PCR methods over the traditional plate count methods, and their wide use for probiotic species and strain identification, PCR methods represent an attractive alternative method for probiotic enumeration, as they enable simultaneous strain-specific qualitative and quantitative detection.

*Bifidobacterium longum* subsp. *longum* is a common bacterium in the gut microbiome of both infants and adults (Oki et al., 2018; Díaz et al., 2021), and strains of this sub species were found to have health benefits such as improving chronic constipation in elderly individuals (Takeda et al., 2023), alleviating glucose intolerance in Type 2 diabetic mice (Kim W. J. et al., 2023), improving cognitive functions in healthy elderly adults (Shi et al., 2023), and reducing perceived stress in healthy adults (Boehme et al., 2023).

Strain *Bifidobacterium longum* subsp. *longum* UABl-14^™^ is a common probiotic strain in probiotic products that was found to support digestive health, modulate bowel functions and increase fibrolytic microbiota in participants with functional constipation when used in combination with other strains (Martoni et al., 2019). Here we present the development and validation of real-time PCR (qPCR) methods for strain specific identification and viable count determination of this important probiotic strain, *Bifidobacterium longum* subsp. *longum* UABl-14^™^.

## Materials and methods

### Reference materials and DNA extraction

In this study, 22 samples of *Bifidobacterium longum* subsp. *longum* strain UABl-14™ were used. Four of these samples were mono-strain samples and 18 were multi-strain samples acquired directly from manufacturers (Table 1). Additionally, reference samples from 30 probiotic strains were included in this study as non-targets (Table 1). The samples were collected from various probiotic manufacturers in USA and Canada. DNA extraction was performed using NucleoSpin Food kit (740945.50, Macherey Nagel, Germany), followed by DNA quantification using Qubit 4.0 FLuorometer (Q33238, Life technologies).

**Table 1:** Target and non-target samples used to confirm the analytical specificity and analytical specificity results of *Bifidobacterium longum* subsp. *longum* UABl-14 strain-specific identification method.

Sample ID	Sample type	Strain	Mean Cq ± SEM *, #
T-1	Target (Mono-strain)	*Bifidobacterium longum* subsp. *longum* UABl-14	22.89 ± 0.08
T-2	Target (Mono-strain)	*Bifidobacterium longum* subsp. *longum* UABl-14	22.87 ± 0.12
T-3	Target (Mono-strain)	*Bifidobacterium longum* subsp. *longum* UABl-14	23.28 ± 0.07
T-4	Target (Mono-strain)	*Bifidobacterium longum* subsp. *longum* UABl-14	22.47 ± 0.04
T-5	Target (Multi-strain)	*Bifidobacterium longum* subsp. *longum* UABl-14	22.55 ± 0.02
T-6	Target (Multi-strain)	*Bifidobacterium longum* subsp. *longum* UABl-14	25.81 ± 0.03
T-7	Target (Multi-strain)	*Bifidobacterium longum* subsp. *longum* UABl-14	26.00 ± 0.04
T-8	Target (Multi-strain)	*Bifidobacterium longum* subsp. *longum* UABl-14	26.14 ± 0.07
T-9	Target (Multi-strain)	*Bifidobacterium longum* subsp. *longum* UABl-14	26.27 ± 0.14
T-10	Target (Multi-strain)	*Bifidobacterium longum* subsp. *longum* UABl-14	25.99 ± 0.08
T-11	Target (Multi-strain)	*Bifidobacterium longum* subsp. *longum* UABl-14	26.39 ± 0.04
T-12	Target (Multi-strain)	*Bifidobacterium longum* subsp. *longum* UABl-14	25.17 ± 0.21
T-13	Target (Multi-strain)	*Bifidobacterium longum* subsp. *longum* UABl-14	25.03 ± 0.23
T-14	Target (Multi-strain)	*Bifidobacterium longum* subsp. *longum* UABl-14	26.06 ± 0.25
T-15	Target (Multi-strain)	*Bifidobacterium longum* subsp. *longum* UABl-14	27.59 ± 0.16
T-16	Target (Multi-strain)	*Bifidobacterium longum* subsp. *longum* UABl-14	25.80 ± 0.04
T-17	Target (Multi-strain)	*Bifidobacterium longum* subsp. *longum* UABl-14	27.79 ± 0.05
T-18	Target (Multi-strain)	*Bifidobacterium longum* subsp. *longum* UABl-14	24.40 ± 0.06
T-19	Target (Multi-strain)	*Bifidobacterium longum* subsp. *longum* UABl-14	23.41 ± 0.15
T-20	Target (Multi-strain)	*Bifidobacterium longum* subsp. *longum* UABl-14	23.47 ± 0.10
T-21	Target (Multi-strain)	*Bifidobacterium longum* subsp. *longum* UABl-14	23.58 ± 0.02
T-22	Target (Multi-strain)	*Bifidobacterium longum* subsp. *longum* UABl-14	28.16 ± 0.03
NT-1	Non-target	*Bifidobacterium animalis* subsp. *lactis* Bi-07	NA
NT-2	Non-target	*Bifidobacterium animalis subsp*. *lactis* UABla-12	NA
NT-3	Non-target	*Bifidobacterium bifidum* Bb-06	NA
NT-4	Non-target	*Bifidobacterium bifidum* HA-132	NA
NT-5	Non-target	*Bifidobacterium bifidum* UABb-10	NA
NT-6	Non-target	*Bifidobacterium breve* Bb-03	NA
NT-7	Non-target	*Bifidobacterium breve* HA-129	NA
NT-8	Non-target	*Bifidobacterium longum* subsp. *infantis* Bi-26	NA
NT-9	Non-target	*Bifidobacterium longum* subsp. *infantis* HA-116	NA
NT-10	Non-target	*Bifidobacterium longum* subsp. *infantis* R0033	NA
NT-11	Non-target	*Bifidobacterium longum* subsp. *longum* Bl-05	NA
NT-12	Non-target	*Bifidobacterium longum* subsp. *longum* HA-135	NA
NT-13	Non-target	*Bifidobacterium longum* subsp. *longum* R0175	NA
NT-14	Non-target	*Lacticaseibacillus casei* Lc-11	NA
NT-15	Non-target	*Lacticaseibacillus casei* UALc-03	NA
NT-16	Non-target	*Lacticaseibacillus paracasei* Lpc-37	NA
NT-17	Non-target	*Lacticaseibacillus paracasei* UALpc-04	NA
NT-18	Non-target	*Lacticaseibacillus rhamnosus* HN001	NA
NT-19	Non-target	*Lacticaseibacillus rhamnosus* Lr-32	NA
NT-20	Non-target	*Lactiplantibacillus plantarum* Lp-115	NA
NT-21	Non-target	*Lactiplantibacillus plantarum* UALp-05	NA
NT-22	Non-target	*Lactobacillus acidophilus* DDS-1	NA
NT-23	Non-target	*Lactobacillus acidophilus* La-14	NA
NT-24	Non-target	*Lactobacillus gasseri* BNR17	NA
NT-25	Non-target	*Lactobacillus gasseri* Lg-36	NA
NT-26	Non-target	*Lactobacillus helveticus* R0052	NA
NT-27	Non-target	*Levilactobacillus brevis* Lbr-35	NA
NT-28	Non-target	*Ligilactobacillus salivarius* Ls-33	NA
NT-29	Non-target	*Limosilactobacillus reuteri* 1E1	NA
NT-30	Non-target	*Limosilactobacillus reuteri* LRC	NA

*SEM: The standard error of the mean.

^#^NA, No amplification.

### Strain-specific real-time PCR oligo design and real-time PCR protocol

UABl-14 strain-specific oligos were designed to amplify a strain specific sequence region that was identified using the sequence-based comparison function in Rapid Annotation using Subsystem Technology (RAST) (Aziz et al., 2008; Overbeek et al., 2014; Brettin et al., 2015). Initially, the genome sequence of UABl-14 was compared to three other *B. longum* strain. The target sequence region identified from RAST was then searched on NCBI GenBank nucleotide collection database using the Basic Local Alignment Search Tool nucleotide function (BLASTn) to confirm the specificity of the identified target region to strain UABl-14. The oligos were designed using PrimerQuest Tool [Integrated DNA Technologies (IDT), Coralville, IA, United States] and were ordered from IDT (Table 2).

**Table 2:** *Bifidobacterium longum* subsp. *longum* UABl-14 strain-specific primer and probe sequences.

Primer/probe	Sequence
Primer F	5′-CATCACACGAGAGAGCACAT-3′
Primer R	5′-CATAGAGAAGCTATCGCCGTATT-3′
Probe	5′-CGCCATCACATGTGCCAATCACAT-3′ (56-FAM and ZEN – 3IABkFQ)

Each real-time PCR reaction consisted of 10 μL of 2x SensiFast Probes Master Mix (BIO-86020, Bioline), 1.8 μL of forward primer (10 μM working solution), 1.8 μL of reverse primer (10 μM working solution), 1.0 μl of hydrolysis probe (5 μM working solution), 1 μL of DNA, and up to 20 μL of molecular biology grade water. The thermal cycling program was denaturation for 5 min at 95°C followed by 40 amplification cycles (for 10 s at 95°C, and for 20 s at 60°C). Positive controls (DNA extracted from a reference sample of UABl-14 and diluted to 1 ng/μl) and negative controls (No Template Controls, NTC) were included in each run and samples were tested in triplicate on Hyris bCUBE.

### Evaluating the specificity and sensitivity of UABl-14 strain-specific assay

To evaluate the specificity of the developed method, real-time PCR was run using 22 target samples (4 mono-strain and 18 multi-strain samples) and 30 non-target samples which included closely related strains such as other *Bifidobacterium longum* strains (Table 1). The same amount of DNA was used from all target and non-target samples. All DNA samples were quantified using Qubit 4.0 Fluorometer, then diluted to 1 ng/μl in molecular biology grade water (Shehata et al., 2019).

To evaluate the sensitivity or limit of detection (LOD), three 10-fold dilution series of DNA, with five dilution points each were used. The dilutions were 10 ng/μl to 0.001 ng/μl, 5 ng/μl to 0.0005 ng/μl and 2 ng/μlto 0.0002 ng/μl (Shehata et al., 2019; Shehata and Newmaster, 2020c). Each dilution point was tested in triplicate using real-time PCR as described above.

### Optimization of viability pre-treatments

A viability dye treatment was used to enumerate viable cells only (Gobert et al., 2018). A viability dye has the ability to cross cell membranes of dead or membrane damaged cells only, and to irreversibly intercalate to DNA upon photoactivation, rendering DNA from dead or membrane damaged cells unreactive in PCR. Multiple concentrations of the viability dye were evaluated to find an effective concentration to inactivate DNA from dead cells as previously described (Shehata and Newmaster, 2021; Shehata et al., 2023). The heat-killed cells were prepared by heating the cells at 95°C for 20 min. PMAxx (40069, Biotium Inc., Hayward, CA, United States) at final concentrations of 0 μM, 50 μM, 100 μM, and 150 μM were tested. The cells and PMAxx were vortexed, followed by incubation at room temperature in the dark for 5 min. Tubes were then incubated in a PhAST BLUE Photoactivation System (GenIUL, Barcelona, Spain) for 15 min. DNA was liberated using bead beating in BeadBug^™^ prefilled tubes (Z763764, Sigma-Aldrich, St. Louis, MO, United States) for 5 min at 3,000 rpm (Hansen et al., 2018; Shehata et al., 2023). The integrity of the DNA from non-heated and heat-killed cells was evaluated by running the DNA from three reference samples T-1, T-2, and T-3 for 10 min on 2% E-gel with SYBR Safe DNA Gel Stain (G720802, Invitrogen), followed by visual inspection of the gel. E-Gel^™^ 1 Kb Plus DNA Ladder (10488090, Invitrogen) was used as a marker. The concentrations of DNA from the same samples were measured using Qubit 4.0 Fluorometer (Q33238, Life technologies). The effectiveness of the viability dye treatment in removing DNA from heat-killed cells was then calculated based on the shift in the Cq values observed with the treatment (Marole et al., 2024).

### Evaluating the reaction efficiency and precision of UABl-14 strain-specific assay

Reaction efficiency, limit of quantification (LOQ), and linear dynamic range were evaluated. Ten-fold serial dilutions were prepared from reference samples at five dilution points each. Each dilution point was tested in triplicate using real-time PCR as described above. Standard curves were established between quantification cycle (Cq) and log genome number. Slopes were calculated from the standard curves using Prism 10 (GraphPad Software, San Diego, CA, United States) and were used to calculate reaction efficiency (Shehata and Newmaster, 2021; Shehata et al., 2023).

Repeatability and reproducibility were evaluated using 3 samples (samples T-1, T-2, and T-3) tested at five dilutions as previously described (Shehata and Newmaster, 2021; Shehata et al., 2023). The analysis was repeated on a different day for repeatability, and on a different bCUBE machine for reproducibility, and the variance was calculated as the relative standard deviation (RSD%).

### Assessing the ability of UABl-14 strain-specific assay in monitoring strain stability in multi-strain finished products during shelf life

The viable counts of strain UABl-14 in 18 multi-strain finished products were determined using UABl-14 strain-specific assay by interpolation from the standard curve. The products were at different expiration dates with 7 products tested post-expiration and 11 products tested pre-expiration dates. All products were stored at room temperature. The viable counts were compared to label claims of viable counts. Additionally, the total counts (viable and dead) of strain UABl-14 were determined using UABl-14 strain-specific assay but eliminating the use of PMAxx.

### Statistical analysis

Prism 10 (GraphPad Software, San Diego, United States) was used for statistical analyses and graphical displays.

## Results

### Strain-specific real-time PCR oligo design

RAST identified a target sequence region in the genome sequence of strain UABl-14, which codes for a hypothetical protein. To confirm that this target sequence region was unique to strain UABl-14, the target sequence region was BLASTn searched on NCBI GenBank in December 2020 and no similarity was found to any sequence in the Nucleotide collection (nr/nt) database. PrimerQuest Tool was used to design primers and a probe to amplify a 94 bp amplicon.

### Evaluating the specificity and sensitivity of UABl-14 strain-specific assay

To confirm the strain specificity of the method, 22 target samples and 30 non-target samples were tested using the developed method (Table 1). All target samples successfully amplified with mean Cq value between 22.47 and 28.16. No amplification was observed from any of non-target samples including other *B. longum* strains (Table 1).

**Figure 1:**
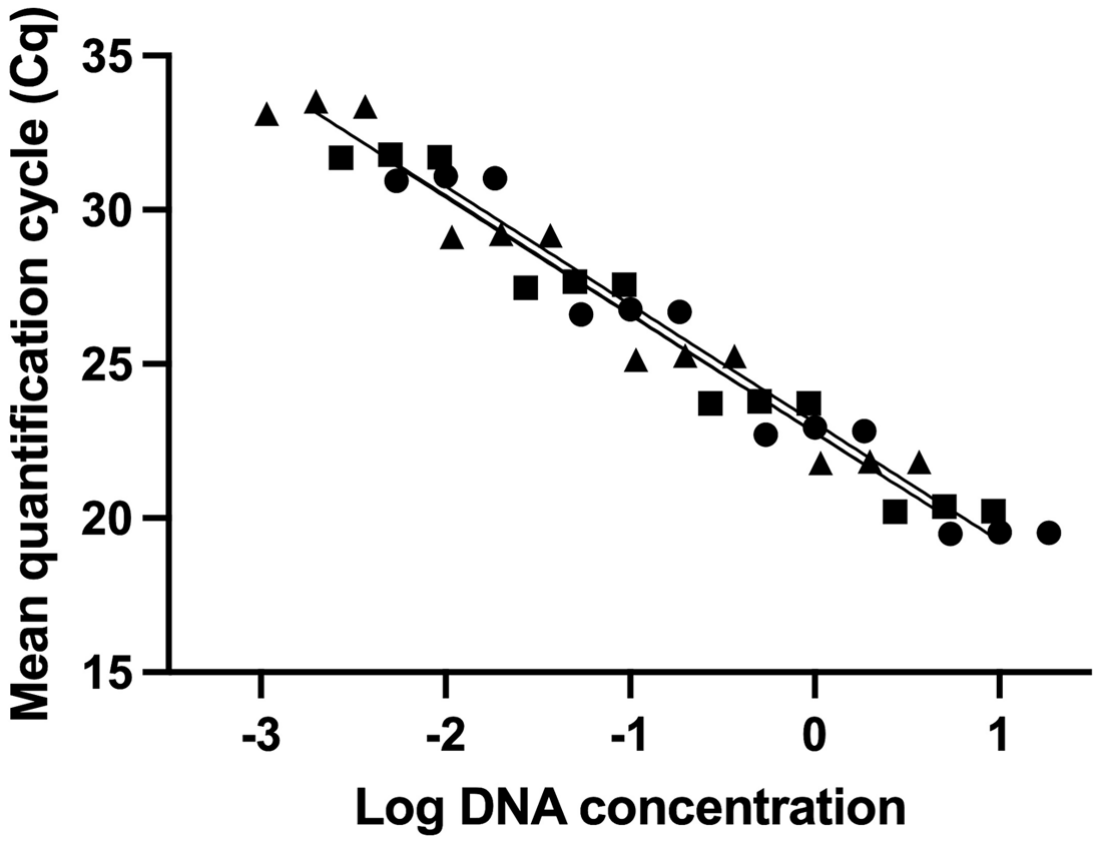
Evaluating the sensitivity of UABl-14 strain-specific assay. Three 10-fold dilution series of DNA were used to establish standard curves. The LOD was 0.002 ng of DNA or 755 copies.

The LOD was determined from standard curves established from three DNA dilution series (5 dilution points each). The LOD was 0.002 ng of DNA or 755 copies (Figure 1).

### Optimization of viability pre-treatments

**Figure 2:**
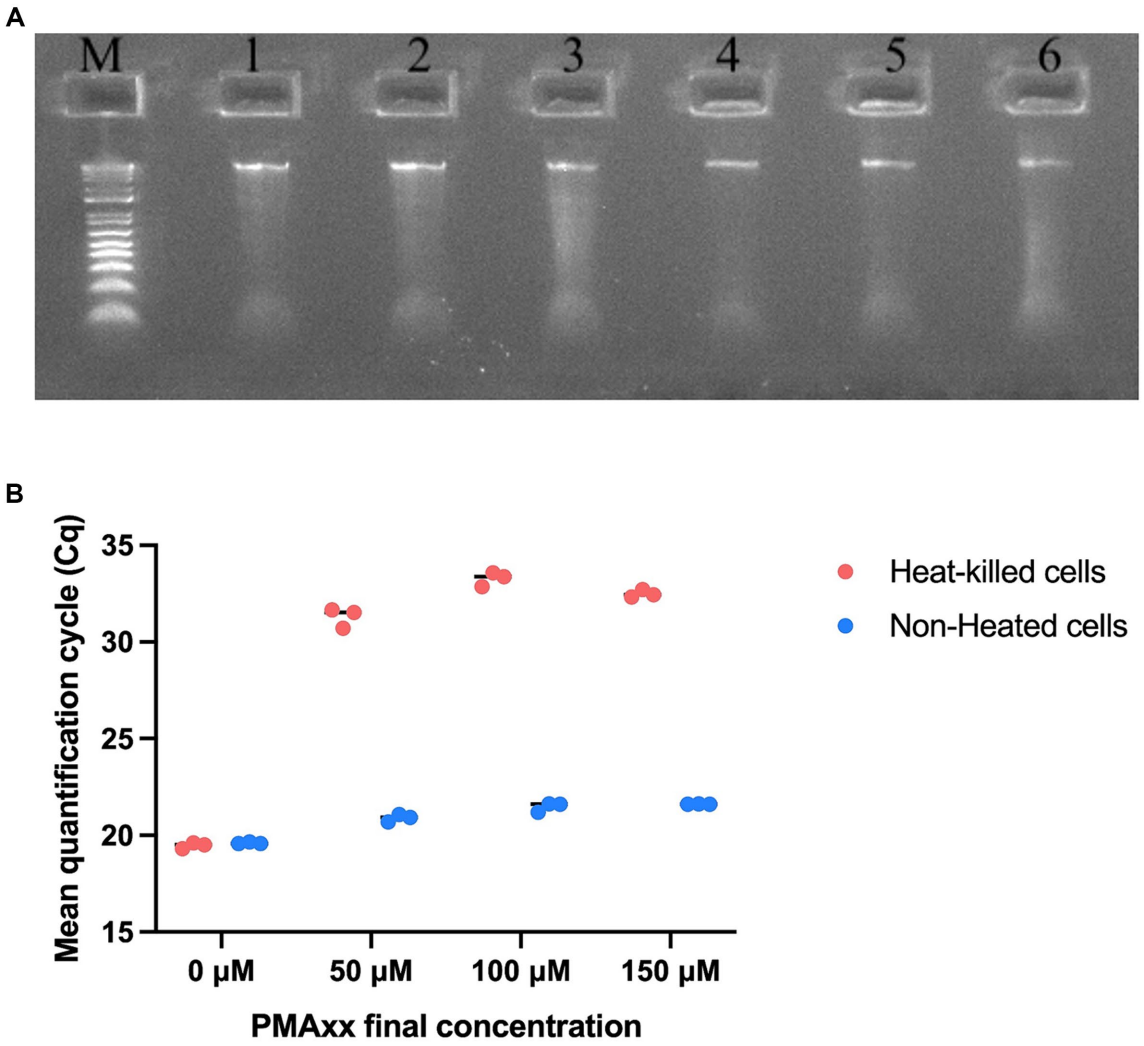
Optimization of viability pre-treatments of UABl-14 strain-specific assay. **(A)** Agarose gel electrophoresis to examine the integrity of the DNA from non-heated and heat-killed cells. M is E-Gel^™^ 1 Kb Plus DNA ladder. Samples 1–3 are the DNA from samples T-1, T-2, and T-3 (non-heated) and samples 4–6 are the DNA from samples T-1, T-2, and T-3 (heat-killed). **(B)** PMAxx viability dye treatments at 0 μM, 50 μM, 100 μM, and 150 μM were evaluated. PMAxx at 50 μM was used as an effective concentration in inactivating DNA from dead cells.

The integrity of the extracted DNA was examined by running the DNA on a gel. DNA extracted from both non-heated and heat-killed cells of samples T-1, T-2, and T-3 showed high integrity (Figure 2A). The DNA concentrations from non-heated cells of samples T-1, T-2, and T-3 were 7 ng/μl, 8 ng/μl, and 8 ng/μl, and from heat-killed cells were 6 ng/μl, 7 ng/μl, and 7 ng/μl. Different concentrations of PMAxx viability dye (0 μM, 50 μM, 100 μM, and 150 μM) were evaluated using non-heated and heat-killed cells to find a concentration that achieved effective inactivation of DNA from dead cells. At 0 μM of PMAxx, non-heated and heat-killed cells showed similar Cq values (19.60 and 19.48, respectively). At 50 μM of PMAxx, non-heated and heat-killed cells showed different Cq values (20.90 and 31.29, respectively). Similar results were observed at 100 μM and 150 μM of PMAxx. At 100 μM of PMAxx, Cq values were 21.47 and 33.27 from non-heated and heat-killed cells, respectively. At 150 μM of PMAxx, Cq values were 21.62 and 32.49 from non-heated and heat-killed cells, respectively (Figure 2B). 50 μM of PMAxx was effective in inactivating dead cells’ DNA from reacting in PCR. This viability dye treatment resulted in a significant shift in Cq value (11.8 cycles), achieving 99.97% removal of DNA from heat-killed cells.

### Evaluating the reaction efficiency and precision of UABl-14 strain-specific assay

**Figure 3:**
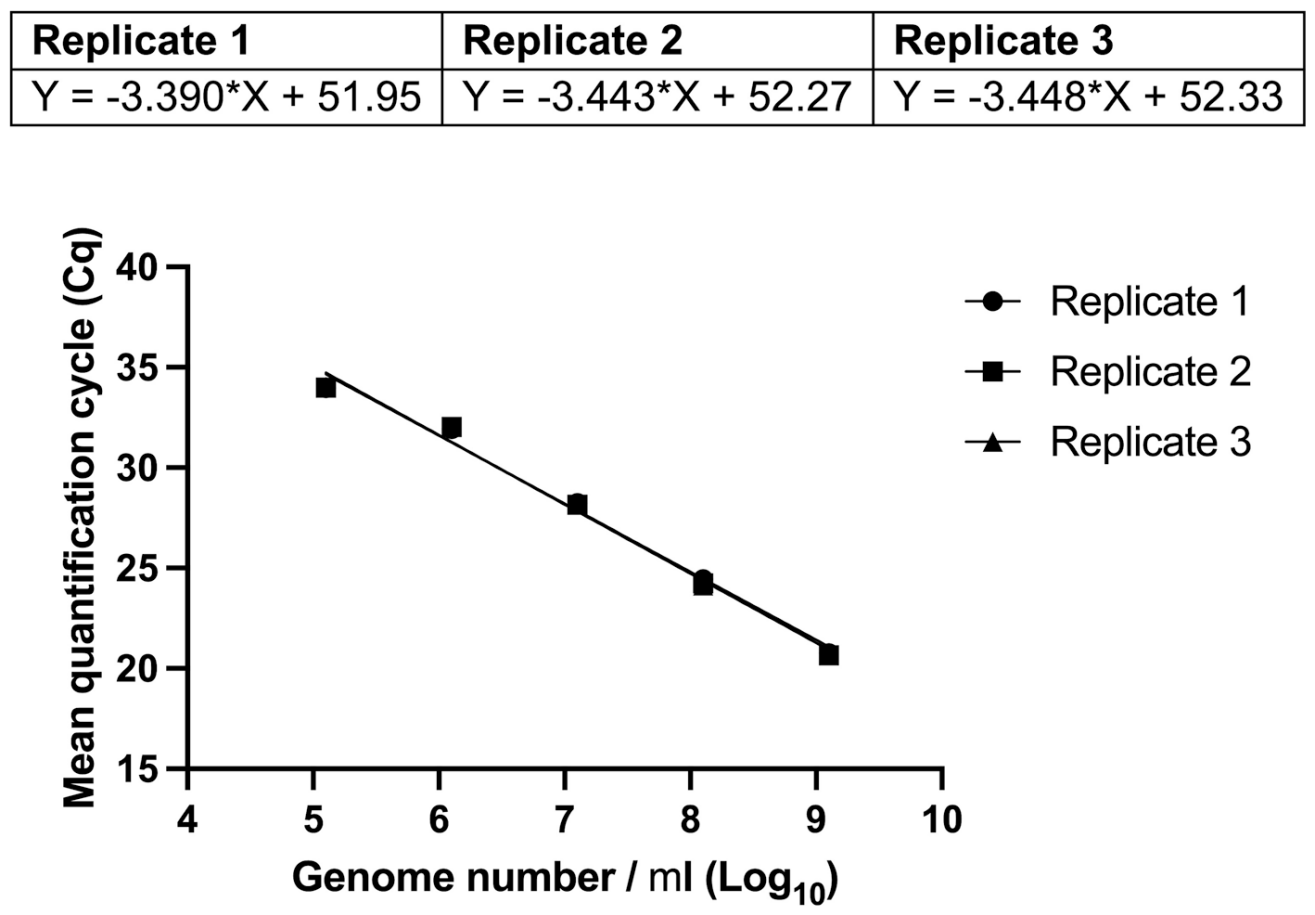
Evaluating the reaction efficiency and precision of UABl-14 strain-specific assay. Reaction efficiency of the UABl-14 strain-specific assay was determined to be 97.2, 95.2, and 95% with *R*^2^ values of 99% and *p* value of 0.0004, 0.0005, and 0.0005 in three replicates.

Reaction efficiency of the UABl-14 strain-specific assay was determined to be 97.2, 95.2, and 95.0% with *R*^2^ values of 99% and *p* value of 0.0004, 0.0005, and 0.0005 in three replicates (Figure 3). The linear dynamic range was 1.3 × 10^2^ to 1.3 × 10^5^ genomes (Figure 3).

**Figure 4:**
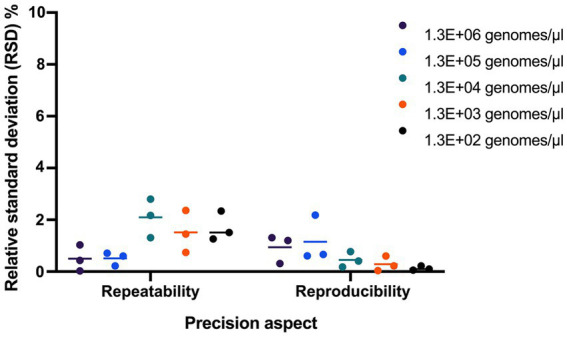
Evaluating the precision of UABl-14 strain-specific assay. Repeatability and reproducibility were evaluated using 3 samples tested at five dilutions. The RSD% for repeatability ranged from 0.71 to 2.36, 0.03 to 1.51, and 0.43 to 2.80, and RSD% for reproducibility ranged from 0.06 to 0.61, 0.10 to 1.20, and 0.04 to 2.18 for the 3 samples.

Repeatability and reproducibility were evaluated using 3 samples tested at five dilutions. The RSD% for repeatability ranged from 0.71 to 2.36, 0.03 to 1.51, and 0.43 to 2.80, and RSD% for reproducibility ranged from 0.06 to 0.61, 0.10 to 1.20, and 0.04 to 2.18 for the 3 samples (Figure 4).

### Assessing the ability of UABl-14 strain-specific assay in monitoring strain stability In multi-strain finished products during storage

**Figure 5:**
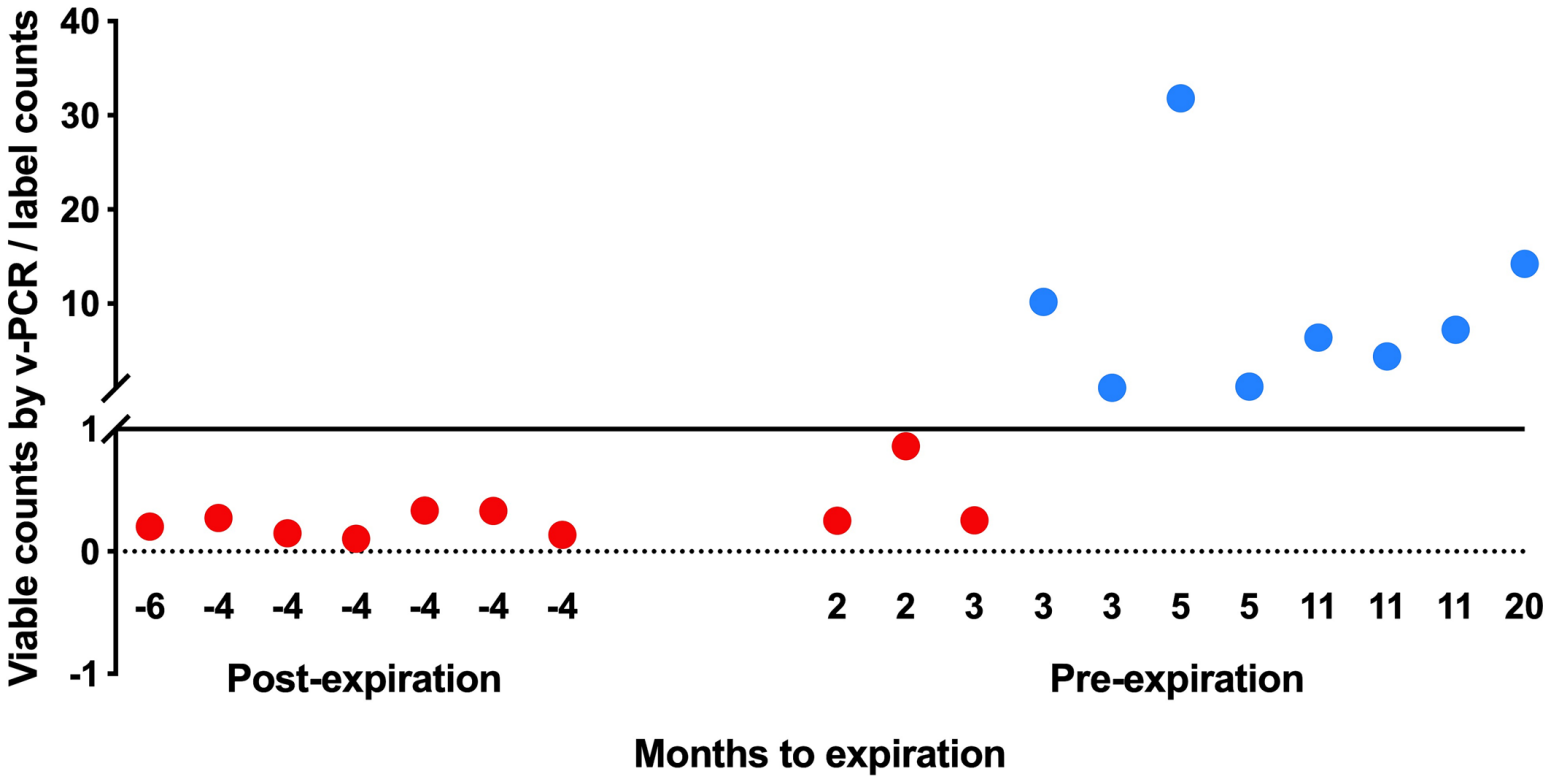
Assessing the ability of UABl-14 strain-specific assay in monitoring strain stability in 18 multi-strain finished products during shelf life. The viable counts were lower than label claims in all 7 products tested post expiration dates and were higher than label claims in products tested pre-expiration dates, with the exception of samples that were within 3 months of expiration.

**Figure 6:**
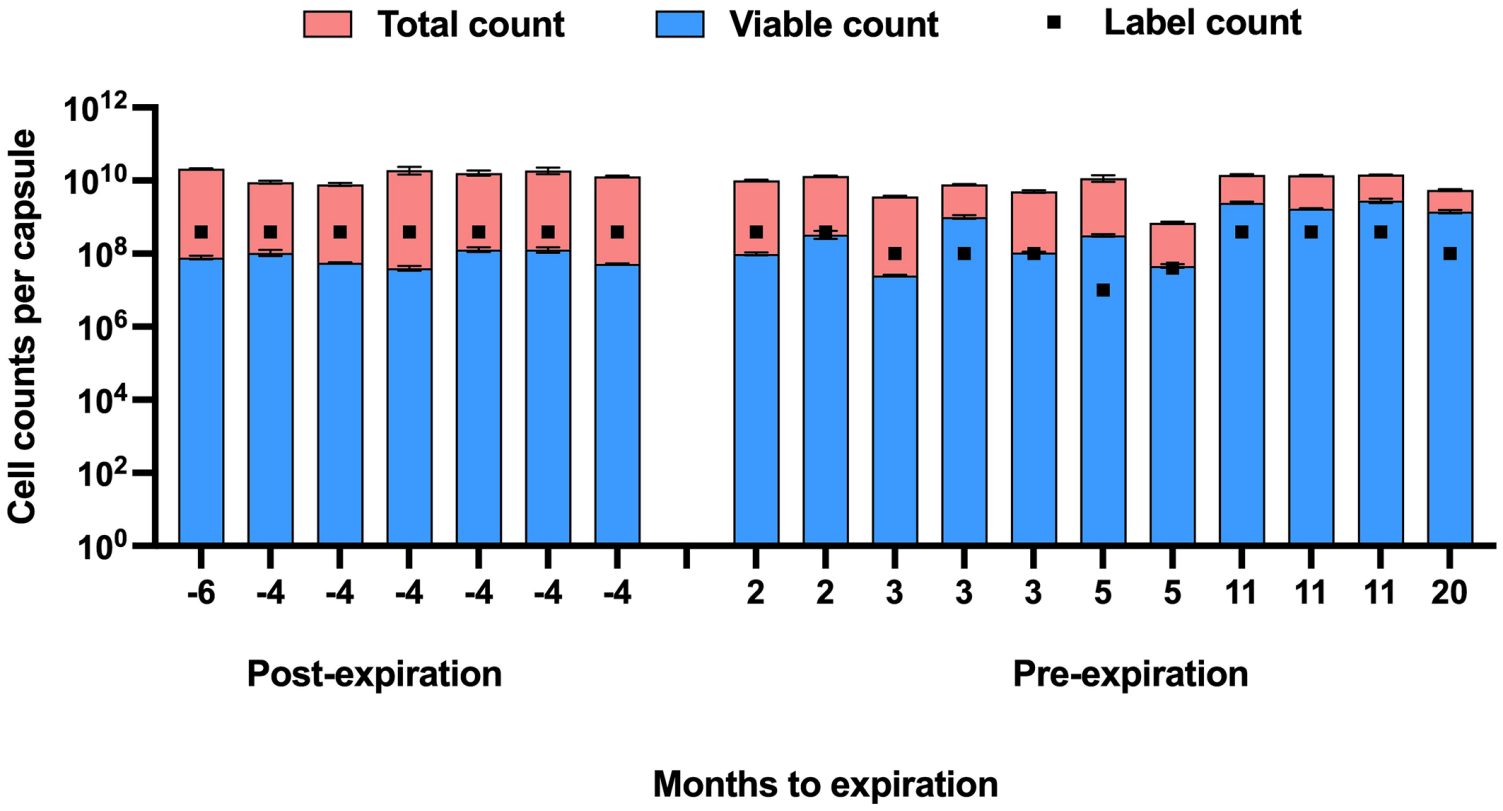
Total counts (viable and dead) and viable counts of strain UABl-14 versus label counts in 18 multi-strain finished products during shelf life. Unlike the viable counts of strain UABl-14, the total counts of strain UABl-14 were consistently higher than label claims in all 18 products.

The viable counts of strain UABl-14 were determined in 18 multi-strain finished products at different expiration dates. The viable counts were lower than label claims in all 7 products tested post expiration dates (Figure 5). The viable counts were higher than label claims in products tested pre-expiration dates except for samples that were within 3 months to expiration (Figure 5). Interestingly, the total counts (viable and dead) of strain UABl-14 were consistently higher than label claims in all 18 products (Figure 6).

## Discussion

Probiotics are sold in food format such as fermented food products as well as in pharmaceutical dosage forms such as capsules and tablets as natural health products or dietary supplements (Health Canada, 2003). The global probiotic market size is growing rapidly, valued at USD 58.17 billion in 2021, and anticipated to reach USD 111.21 billion in 2030 (Grand-View-Research-Inc, 2022). With the expanding market size, multiple reports have shown failure of probiotic products to meet label claims, observed as strain substitution, missing strains, presence of undeclared strains or lower viable counts compared to label claims during shelf life and before expiration dates (Morovic et al., 2016; Patro et al., 2016; Kolaček et al., 2017; Shehata and Newmaster, 2020a,b). This label non-compliance can result in partial or complete loss of efficacy (Tripathi and Giri, 2014; Kolaček et al., 2017; Sánchez et al., 2017; Jackson et al., 2019). Thus, analytical methods that support product authentication via confirming label information about product content is extremely important (Fusco et al., 2023).

*B. longum* subsp. *longum* UABl-14 is a common probiotic strain in probiotic products that was proven to support digestive health (Martoni et al., 2019). However, to the best of our knowledge, there are no available methods to achieve strain-specific identification and enumeration of this strain. In this study, real-time PCR based methods for strain-specific identification and enumeration of strain UABl-14 were developed and validated to facilitate the quality assurance of probiotic products that contain this strain.

A strain-specific identification and/or enumeration method requires robust bioinformatic analyses of genome sequences to confirm strain specificity, as well as extensive validation to ensure accurate and precise performance. Bioinformatic analyses identified a unique sequence region in the genome of strain UABl-14. The sequence region showed no similarity to any sequence in the Nucleotide collection database in NCBI GenBank. Primers and a hydrolysis probe were designed to target this unique sequence region. The primers and probe were validated for use in strain-specific identification and enumeration methods. The specificity of the UABl-14 strain-specific assay was evaluated in qPCR where the assay successfully amplified all 22 target samples, which included mono-strain and multi-strain samples. Thirty non-target samples were used in specificity evaluation which included multiple strains of lactobacilli and *Bifidobacterium,* and included, other strains of *B. longum* such as *Bifidobacterium longum* subsp. *infantis* strains Bi-26, HA-116, and R0033 and *Bifidobacterium longum* subsp. *longum* strains Bl-05, HA-135, and R0175 to confirm strain level specificity (Table 1). No amplification was observed from any non-target strains. It is important to note that these non-target strains are commercialized probiotic strains available and common in the market in finished probiotic products. The results confirmed that the assay is strain specific to strain UABl-14 which means the assay will correctly identify strain UABl-14 only. The results also confirmed that the assay works well with both mono-strain and multi-strain samples.

The sensitivity of the UABl-14 strain-specific assay was also evaluated in qPCR. Sensitivity or the LOD is the lowest amount of the target that an assay can detect (Bustin et al., 2009). Standard curves were established and the LOD was determined to be 0.002 ng of DNA (Figure 1). Thus, the assay proved to be highly sensitive, which means the assay is applicable to multi-strain blends and products in which strain UABl-14 is present at low abundance.

The UABl-14 strain-specific assay was further validated for quantitative use for the enumeration of strain UABl-14. To enumerate viable cells only, the assay was used with PMAxx viability dye, a DNA-intercalating dye that inactivates DNA from dead cells. The viability dye treatment is known to vary between strains and thus optimization for each target strain is required (Kiefer et al., 2020). Optimization of PMAxx viability dye treatment with strain UABl-14 showed that 50 μM of PMAxx was effective in inactivating DNA from dead cells from reacting in PCR, achieving 99.97% removal of DNA from heat-killed cells (Figure 2B). Previous studies reported optimal final concentrations of PMA that ranged from 25 μM to 100 μM (Gobert et al., 2018; Hansen et al., 2018; Scariot et al., 2018; Shehata and Newmaster, 2021; Shehata et al., 2023).

A very important parameter to be considered when evaluating a quantitative assay is the reaction efficiency, with the ideal reaction efficiency ranging between 90 and 110% with *R*^2^ values ≥ 0.98 (Broeders et al., 2014). Reaction efficiency values of the UABl-14 strain-specific assay were 97.2, 95.2, and 95.0% and *R*^2^ value was 99% in all three replicates (Figure 3). The linear dynamic range covered four dilutions points (Figure 3). An ideal dynamic range covers 5 to 6 dilutions, with a minimum of three dilutions (Bustin et al., 2009). Thus, the UABl-14 strain-specific assay has high efficiency and adequate linear dynamic range.

The repeatability and reproducibility of the UABl-14 strain-specific assay were evaluated. The RSD% for repeatability using three samples tested at five dilutions was below 2.80, and RSD% for reproducibility using three samples tested at five dilutions ranged was below 2.18 (Figure 4). The results indicate that the UABl-14 strain-specific assay is highly precise, since the acceptable value for repeatability and reproducibility is below 25% (Broeders et al., 2014).

The UABl-14 strain-specific assay was evaluated for the ability to monitor strain stability in multi-strain finished products during storage by testing 18 multi-strain finished products at different expiration dates. The methods showed variable viable and total (viable and dead) counts of strain UABl-14 in finished products tested at different expiration dates (Figures 5, 6). Viability of probiotic strains is expected to decline during storage, the decline rate varying with storage conditions such as temperature and moisture levels (Tripathi and Giri, 2014). Improving strain stability during shelf life of probiotic products is a major challenge in the probiotic industry (Morovic et al., 2016). Probiotic products are expected to meet label claims of viable count until expiration dates to maintain efficacy. Thus, methods that enable strain-specific monitoring of stability during shelf life is of great importance.

Because the probiotic products that were used in the stability monitoring experiment were multi-strain products, it was not possible to compare the viable counts to plate counts. Nonetheless, plate count and viability PCR measure viability differently where plate count methods rely on cultivability while viability PCR relies on membrane integrity as a measure of viability (Boyte et al., 2023). Previous studies have reported discrepancies in viable counts determined using culture-dependent versus culture-independent methods, especially following storage (Fiore et al., 2020; Wendel, 2022; Shehata et al., 2023). This may be attributed to the fact that cell cultivability declines faster than membrane integrity, and to the portion of cells that exist in a VBNC state (Foglia et al., 2020). Since VBNC cells are considered probiotics, viable counts determined using culture-independent methods would be more accurate compared to culture-dependent methods (Foglia et al., 2020).

## Conclusion

The real-time PCR methods developed and validated for strain-specific identification and viable count determination of strain UABl-14 are strain-specific, highly sensitive and enable the enumeration of VBNC cells. Thus, the methods offer a significant advancement in viable count determination over the traditional plate count method. The methods allow for simultaneous and cost-effective analyses, serving the dual purpose of identification and enumeration of strain UABl-14 in mono-strain as well as in multi-strain finished products to facilitate quality control measures for efficacious and compliant probiotic products.

## Data availability statement

The original contributions presented in the study are included in the article/supplementary material, further inquiries can be directed to the corresponding author.

## Author contributions

HS: Writing – review & editing, Writing – original draft, Visualization, Validation, Supervision, Software, Methodology, Investigation, Formal analysis, Conceptualization. BH: Writing – review & editing, Methodology, Investigation. SN: Writing – review & editing, Supervision, Resources, Project administration, Conceptualization.
